# A Wrist Brace with Integrated Piezoelectric Sensors for Real-Time Biomechanical Monitoring in Weightlifting

**DOI:** 10.3390/mi16070775

**Published:** 2025-06-30

**Authors:** Sofia Garcia, Ethan Ortega, Mohammad Alghamaz, Alwathiqbellah Ibrahim, En-Tze Chong

**Affiliations:** 1 UT Tyler Academy, The University of Texas at Tyler, 3900 University Blvd., Tyler, TX 75799, USA; sofia_garcia@uttia.org (S.G.); ethan_ortega@uttia.org (E.O.); echong@uttia.org (E.-T.C.); 2 Department of Mechanical Engineering, The University of Texas at Tyler, 3900 University Blvd., Tyler, TX 75799, USA; malghamaz@patriots.uttyler.edu

**Keywords:** piezoelectric, energy harvesting, wrist brace, health monitoring, biomechanical calibration

## Abstract

This study presents a self-powered smart wrist brace integrated with a piezoelectric sensor for real-time biomechanical monitoring during weightlifting activities. The system was designed to quantify wrist flexion across multiple loading conditions (0 kg, 0.5 kg, and 1.0 kg), leveraging mechanical strain-induced voltage generation to capture angular displacement. A flexible PVDF film was embedded within a custom-fitted wrist brace and tested on male and female participants performing controlled wrist flexion. The resulting voltage signals were analyzed to extract root-mean-square (RMS) outputs, calibration curves, and sensitivity metrics. To interpret the experimental results analytically, a lumped-parameter cantilever beam model was developed, linking wrist flexion angles to piezoelectric voltage output based on mechanical deformation theory. The model assumed a linear relationship between wrist angle and induced strain, enabling theoretical voltage prediction through simplified material and geometric parameters. Model-predicted voltage responses were compared with experimental measurements, demonstrating a good agreement and validating the mechanical-electrical coupling approach. Experimental results revealed consistent voltage increases with both wrist angle and applied load, and regression analysis demonstrated strong linear or mildly nonlinear fits with high $R^{2}$ values (up to 0.994) across all conditions. Furthermore, surface plots and strain sensitivity analyses highlighted the system’s responsiveness to simultaneous angular and loading changes. These findings validate the smart wrist brace as a reliable, low-power biomechanical monitoring tool, with promising applications in injury prevention, rehabilitation, and real-time athletic performance feedback.

## 1. Introduction

Injuries related to poor wrist biomechanics remain a significant concern in both athletic and occupational settings. Among athletes, particularly those engaged in strength training and resistance exercises, the wrist joint is often subjected to substantial biomechanical loads during flexion, extension, and deviation. Incorrect technique or overloading can lead to repetitive strain injuries (RSIs), tendinitis, or ligament damage, many of which have long recovery times and can significantly impair performance [1,2,3]. Continuous monitoring and real-time feedback on wrist motion, particularly during strenuous activities such as weightlifting, present a promising avenue for injury prevention and biomechanical optimization.

Weightlifting, while recognized for its numerous benefits in improving musculoskeletal health, bone density, and metabolic function, also poses substantial injury risks when executed with improper form [4]. It has been widely adopted not only by athletes but also in general fitness, rehabilitation, and occupational therapy contexts for enhancing muscle strength and functional capacity [5,6]. However, repetitive or excessive loading, especially in the absence of proper technique, can result in a range of upper extremity injuries. The wrist joint is particularly vulnerable due to its complex structure and role in load transmission, making it susceptible to conditions such as tendonitis, ligament sprains, and carpal instability [7]. Studies have identified wrist hyperextension and ulnar deviation as common biomechanical errors associated with increased strain and injury incidence, particularly in exercises involving overhead pressing or gripping heavy weights [8,9]. Improper alignment during repetitive movement not only increases stress on the radiocarpal and midcarpal joints but also interferes with optimal force production and movement efficiency.

Traditional approaches to injury prevention in weightlifting rely heavily on coaching feedback, visual observation, and post-hoc video analysis. While effective for trained athletes in supervised environments, these methods are inherently limited in their ability to provide real-time correction and are often inaccessible to casual or recreational lifters who make up the majority of gym-goers [10,11]. Additionally, self-perception of form is often inaccurate, particularly under fatigue, making subjective feedback an unreliable tool for injury prevention [12]. To address these limitations, there has been growing interest in the integration of wearable technologies capable of real-time biomechanical monitoring. These systems aim to quantify joint angles, kinematic patterns, and muscular loads, offering objective data that can be used to detect unsafe movement patterns and guide corrective interventions [13,14,15]. Wearable devices equipped with inertial sensors, force transducers, or piezoelectric elements have been used to assess lifting technique, joint range of motion, and dynamic load transfer across multiple joints, providing valuable insights for training, rehabilitation, and injury prevention applications.

Recent advancements in wearable rehabilitation technologies have increasingly emphasized the integration of novel materials and self-powered sensing strategies to enable more effective biomechanical monitoring. In particular, two-dimensional (2D) materials have demonstrated excellent potential in rehabilitation medicine due to their superior mechanical flexibility, conductivity, and biocompatibility [16,17]. These materials are enabling the next generation of highly conformable, lightweight, and multifunctional sensing platforms. Simultaneously, self-powered sensors based on piezoelectric and triboelectric principles are gaining traction for their ability to operate without external power sources, thus enhancing device autonomy and simplifying wearable integration [18,19]. These emerging technologies are paving the way toward intelligent, real-time rehabilitation devices that can capture subtle biomechanical cues, provide feedback, and promote adaptive recovery strategies. The present work aligns with this vision by developing a PVDF-based piezoelectric wrist brace that combines experimental validation with theoretical modeling to enable real-time, load-sensitive biomechanical monitoring.

Recent advances in wearable sensing have focused on integrating flexible electronics into garments or accessories to enable real-time motion tracking and physiological monitoring. These systems commonly incorporate technologies such as inertial measurement units (IMUs), strain gauges, optical fibers, and capacitive sensors to capture biomechanical data, including posture, joint angles, and muscle activity [20,21,22]. The development of flexible electronics and e-textiles has facilitated the miniaturization and conformability of such systems, allowing close adherence to the skin or seamless integration into clothing with minimal discomfort or interference [23,24].

Despite these advancements, a persistent limitation remains: most wearable sensors depend on batteries or wired power sources, which impose constraints related to device bulk, maintenance, and long-term usability [25,26,27,28,29]. The need for frequent recharging or battery replacement not only reduces practicality but also increases environmental impact. For continuous, long-term health monitoring, particularly in remote, clinical, or athletic settings, a self-sustaining power supply is essential to ensure uninterrupted operation.

Furthermore, in rehabilitation and human–machine interface applications, polyvinylidene fluoride (PVDF) has emerged as a promising material due to its flexibility, biocompatibility, and electromechanical responsiveness. Prior studies have investigated PVDF-based strain sensors for capturing complex deformations and body movements with high sensitivity and durability [30,31,32,33,34]. These works highlight the relevance of piezoelectric sensing as a low-power, highly responsive solution for continuous human motion tracking, motivating the present study on PVDF-integrated wrist sensing for real-time feedback during physical activities such as weightlifting.

To address these challenges, there has been growing interest in integrating energy harvesting mechanisms into wearable devices, particularly those based on piezoelectric, triboelectric, or thermoelectric principles [35,36,37,38,39,40,41]. Piezoelectric materials convert mechanical deformation into electrical energy, making them ideal for both sensing and powering low-energy electronics. PVDF (polyvinylidene fluoride), in particular, has demonstrated promise in wearable applications due to its flexibility, durability, and high sensitivity to strain [42]. Several studies have explored the integration of piezoelectric films in motion-detecting wearables for gait analysis, joint angle monitoring, and posture correction [43,44,45,46,47]. For instance, Lee et al. developed a self-powered piezoelectric sensor for monitoring joint angles during knee extension exercises [44]. Similarly, Kim et al. proposed a hybrid triboelectric–piezoelectric sensor embedded into a sleeve for elbow monitoring during physical therapy [45]. The dual function of piezoelectric materials, as both energy harvesters and sensors, opens exciting possibilities for developing lightweight, wireless, and sustainable wearable systems. Their incorporation into real-time biomechanics monitoring platforms represents a significant leap toward autonomous healthcare solutions, particularly for applications in rehabilitation, sports science, and occupational safety.

Despite significant progress in wearable piezoelectric technologies, few studies have focused on the wrist joint under weight-bearing conditions, a critical gap given its biomechanical complexity and susceptibility to injury during resistance training. Most existing systems are either limited to qualitative motion detection or require external power and bulky acquisition units. To address these limitations, this study presents the design, calibration, and evaluation of a self-powered, lightweight smart wrist brace integrated with flexible piezoelectric sensors. The system captures wrist kinematics under three loading conditions (0 kg, 0.5 kg, and 1 kg), translating mechanical deformation into measurable voltage signals without external power. While the protocol aimed to include repeated trials per angle, data were collected from single-trial recordings due to practical constraints. Nonetheless, the results establish a strong foundation for future development of wearable biomechanical monitors and contribute to the advancement of injury-prevention technologies in athletic and rehabilitation settings.

## 2. Materials and Methods

### 2.1. Smart Wrist Brace Fabrication and Sensor Integration

The smart wrist brace was fabricated in-house using Polyester Bridal Solid Silky Satin Fabric, selected for its softness, flexibility, and form-fitting properties. The material was tailored into a glove-like design that comfortably conforms to the user’s hand and wrist, enabling unrestricted joint motion. A custom-fitted pocket was sewn into the wrist region of the glove, an anatomically relevant area that experiences substantial mechanical strain during flexion and extension.

A single piezoelectric sensor was inserted into this pocket to capture wrist motion. The sensing element consisted of a polyvinylidene fluoride (PVDF) strip, chosen for its high strain sensitivity, mechanical durability, and flexibility. The PVDF film (dimensions: approximately 50 mm × 10 mm × 52 μm) was encapsulated in protective plastic to maintain electrical insulation and structural stability. The pocket placement ensured direct coupling between wrist deformation and the sensor. As the wrist flexed or extended, mechanical strain was transferred to the PVDF, producing a voltage output via the direct piezoelectric effect.

### 2.2. Experimental Setup and Signal Acquisition

**Figure 1:**
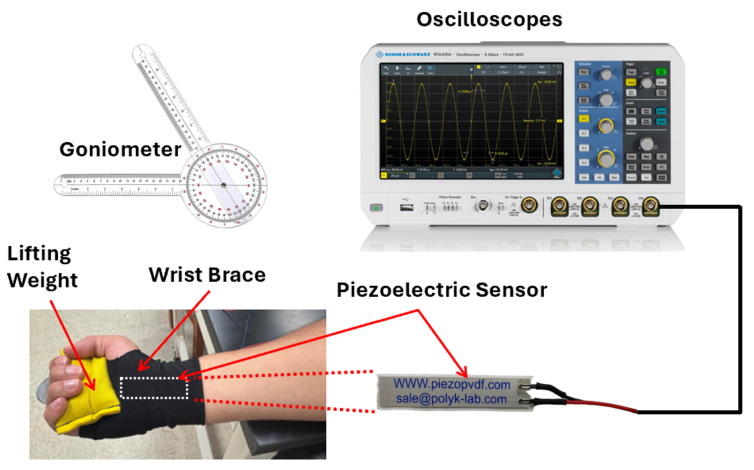
Schematic of the experimental setup showing the custom-fabricated smart wrist brace with integrated piezoelectric sensor, goniometer for angular calibration, and oscilloscope for real-time voltage signal monitoring during wrist movement under load. The white dashed line serves only as a visual indicator of the piezoelectric sensor’s location within the wrist brace.

The smart wrist brace described above was connected to a data acquisition setup, as illustrated in Figure 1. During testing, the user wore the brace and performed controlled wrist flexion and extension movements while holding a lightweight dumbbell to simulate real-world loading conditions. These tests were conducted under three distinct loading scenarios: 0 kg (no load), 0.5 kg, and 1 kg.

To establish the relationship between wrist angle and voltage output, angular calibration was performed using a manual goniometer. Prior to each voltage recording, the goniometer was manually aligned with the wrist brace to set and confirm target angles ranging from 0° to 60° in 10° increments. At each position, the user maintained a brief hold while the output voltage from the piezoelectric sensor was measured. This enabled calibration of mechanical deformation against sensor response under varying loads.

The sensor was wired to a digital oscilloscope (Siglent SDS1202X-E, Siglent Technologies Co., Ltd., Solon, OH, USA), which recorded and visualized the voltage waveforms generated in real time. These waveforms provided immediate insight into the dynamic strain on the sensor at different joint angles. The oscilloscope functioned as the primary data acquisition tool during calibration, enabling precise monitoring of signal magnitude and consistency. The collected data was later exported and processed in MATLAB-R2024b for further analysis.

Testing was performed by one male and one female, both 18 years old and classified as healthy young adults. This dual-subject approach was adopted to account for potential variability in wrist biomechanics across individuals of different sexes. Each participant wore the smart wrist brace and followed the same experimental procedure, including angular calibration and controlled wrist movements under three load conditions (0 kg, 0.5 kg, and 1 kg). The use of consistent age and body region ensured a uniform testing baseline while still allowing preliminary assessment of subject-to-subject variation in piezoelectric output. All tests were performed in a controlled laboratory environment, and the brace was re-adjusted for proper fit between subjects to ensure consistent sensor placement and contact.

## 3. Results and Discussion

### 3.1. Measurement Data

**Figure 2:**
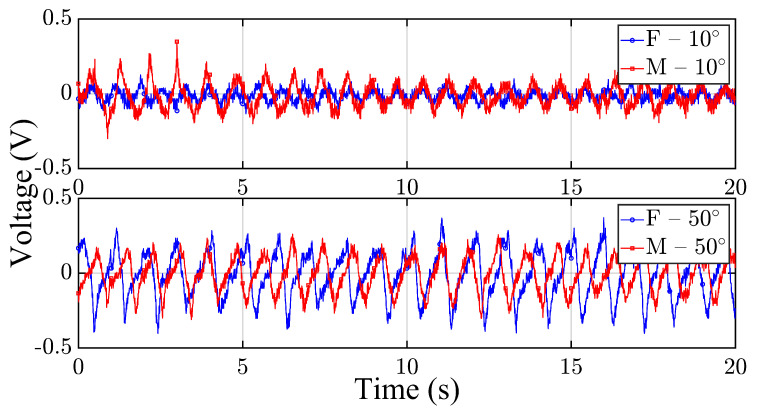
Sample of the time-domain voltage signals under no loading conditions from male and female participants at $10^{{}^{\circ}}$ wrist angle (**top**) and $50^{{}^{\circ}}$ wrist angle (**bottom**).

Figure 2 presents representative time-domain voltage signals recorded from the smart wrist brace during wrist flexion-extension cycles for both participants at two selected joint angles: $10^{{}^{\circ}}$ (top subplot) and $50^{{}^{\circ}}$ (bottom subplot). The female and male signals are shown in blue and red, respectively, with distinct markers (circles and squares) to enhance visual separation. All signals were time-aligned to start at t=0 s to allow for direct comparison of waveform evolution and dynamic response.

At $10^{{}^{\circ}}$, both participants demonstrated relatively low-amplitude signals with consistent rhythmic patterns corresponding to periodic wrist motion. The voltage outputs reflect minor mechanical deformation of the piezoelectric sensor due to shallow angular flexion, which is expected at lower joint angles. The signal shape in both traces is comparable, although the male participant exhibited slightly higher peak-to-peak amplitude, which may be attributed to differences in muscle tension or joint stiffness.

At $50^{{}^{\circ}}$, both participants generated significantly higher voltage outputs, consistent with the increased strain experienced by the wrist brace at larger flexion angles. The waveform for the female participant displayed sharper peaks and greater variability, while the male participant’s waveform appeared more uniform and periodic. This contrast may suggest differences in motion control strategy, wrist biomechanics, or force distribution during repeated wrist cycles. Notably, both participants maintained distinguishable, load-sensitive patterns, indicating that the smart brace effectively captures user-specific mechanical responses and angular dependence.

In the following sections, bar plots are employed to present the experimental results, providing a clearer and more intuitive visualization of the voltage responses across different wrist angles and loading conditions.

### 3.2. No Load Condition

To establish a baseline for wrist flexion without external resistance, voltage responses from the integrated piezoelectric sensors were measured under a no-load condition for both participants. The participants, one female and one male, performed repeated wrist flexion movements at six target angles: $10^{{}^{\circ}}$, $20^{{}^{\circ}}$, $30^{{}^{\circ}}$, $40^{{}^{\circ}}$, $50^{{}^{\circ}}$, and $60^{{}^{\circ}}$. For each angle, the user attempted to reach the designated posture multiple times during a continuous data recording session. The RMS voltage was extracted from each angle’s corresponding time-domain signal to quantify the sensor output.

**Figure 3:**
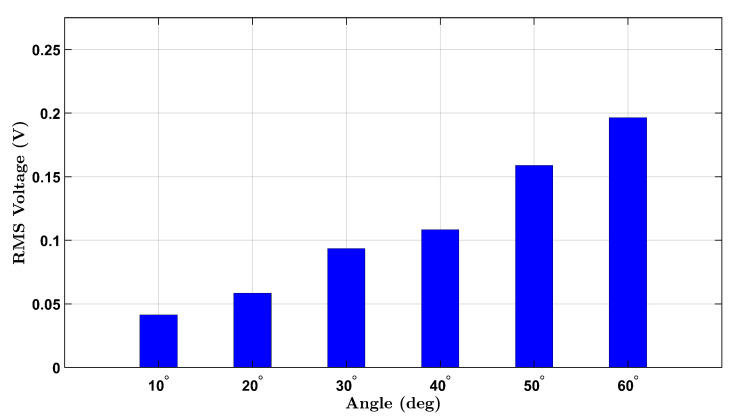
No-load RMS voltage response of the female participant across six wrist flexion angles.

Figure 3 illustrates the results for the female participant, where a progressive increase in voltage is observed with increasing angle, consistent with increased wrist strain. Notably, a nonlinear response appears beyond $40^{{}^{\circ}}$, likely reflecting the cumulative deformation of the piezoelectric layer under higher flexion.

**Figure 4:**
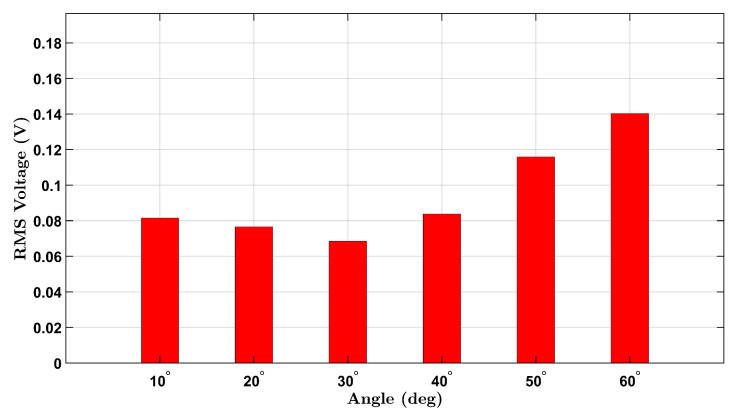
No-load RMS voltage response of the male participant across six wrist flexion angles.

Similarly, the male participant’s results are shown in Figure 4. A comparable upward trend is seen across the six angles, with slightly higher RMS values overall. This may reflect differences in wrist size, muscle stiffness, or motion dynamics between participants. The observed voltage trends align with the expected behavior of piezoelectric materials under mechanical bending and demonstrate the device’s capability to detect strain amplitude through voltage generation.

A non-monotonic voltage trend was observed in the male participant’s no-load condition, where the RMS voltage initially decreased before increasing with angle. This behavior is likely due to biomechanical and experimental variability, including slight inconsistencies in wrist posture and minor brace shifting during low-angle flexion. In contrast, the female participant’s brace maintained better strain coupling, resulting in a more consistent response. These differences diminished at larger angles where strain becomes more uniformly transmitted to the sensor.

These no-load findings provide a comparative foundation for evaluating the sensor’s behavior under additional mass loading and form the basis for future normalization and calibration efforts.

To further analyze the sensor response under no-load conditions, a calibration model was developed to relate RMS voltage output to wrist flexion angles.

**Figure 5:**
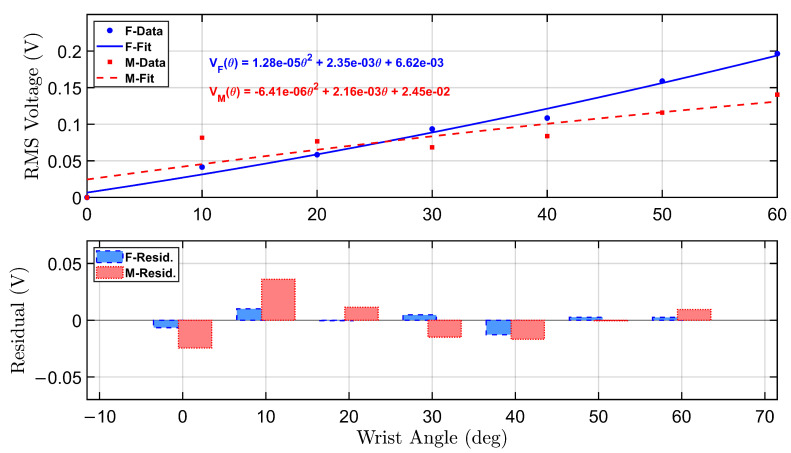
Calibration curves and residual error analysis for female and male participants under no-load condition. The top subplot illustrates the second-order polynomial fits for the relationship between wrist angle and RMS voltage. The bottom subplot presents the residuals between actual and predicted RMS voltages across tested angles.

Figure 5 presents the calibration and residual analysis for both female and male participants under the no-load condition. The top panel displays second-order polynomial fits of the RMS voltage as a function of wrist angle, derived from empirical data collected during repeated flexion trials targeting angles from $0^{{}^{\circ}}$ to $60^{{}^{\circ}}$.

The female participant’s calibration curve is shown in solid blue, with a corresponding equation annotated in the upper right quadrant. The fitted curve is given by:

$$V_{F}\left(\theta \right)=1.28\times 10^{-5}\theta^{2}+2.35\times 10^{-3}\theta +6.62\times 10^{-3}$$

Similarly, the male participant’s calibration curve is plotted in dashed red, described by:

$$V_{M}\left(\theta \right)=-6.41\times 10^{-6}\theta^{2}+2.16\times 10^{-3}\theta +2.45\times 10^{-2}$$

Both curves reveal an increasing trend in RMS voltage with wrist angle, though the female profile demonstrates a steeper response, indicating greater voltage sensitivity across the range of motion. This is consistent with higher sensor output amplitudes observed in the female recordings, particularly at higher angles.

The bottom panel displays the residual errors for both polynomial fits. The residuals represent the deviation between the actual RMS voltage and the predicted value from the calibration equation. Residual bars for the female participant (blue, dashed border) and male participant (red, dotted border) indicate overall good model agreement, with most residuals tightly distributed around zero. The female model shows slightly larger residual fluctuations at mid-range angles, while the male model residuals are more variable at the low and high extremes.

These calibration curves serve as reliable transfer functions for mapping sensor voltage to wrist flexion angle in real-time applications. The residual analysis supports the validity of using second-order polynomials for both participants and highlights individual differences that can inform future model personalization strategies.

### 3.3. 0.5 kg Load Condition

**Figure 6:**
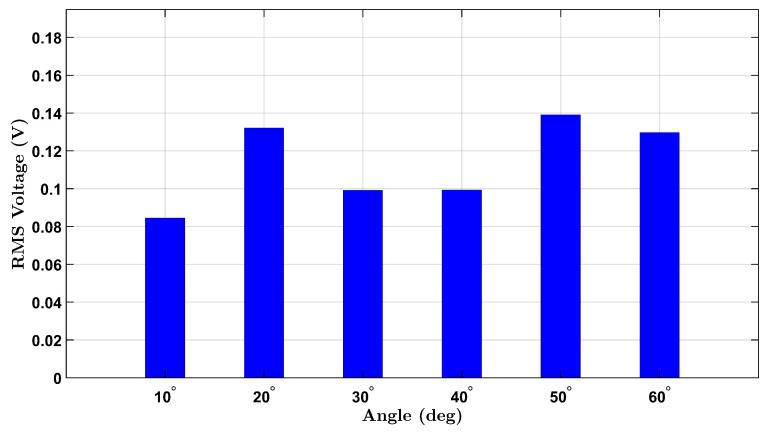
RMS voltage response of the female participant at 0.5 kg load across six wrist flexion angles.

**Figure 7:**
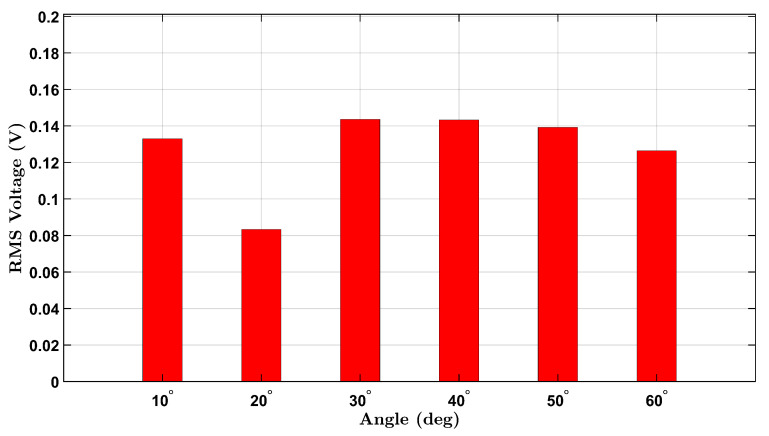
RMS voltage response of the male participant at 0.5 kg load across six wrist flexion angles.

To evaluate the influence of external loading on piezoelectric voltage response, wrist flexion tests were conducted under a 0.5 kg load for both participants. The root-mean-square (RMS) voltage values were extracted from time-domain signals corresponding to six discrete wrist flexion angles. The resulting data are visualized in Figure 6 and Figure 7.

As illustrated in the figures, both participants showed a general increase in RMS voltage as the wrist flexion angle increased from $10^{{}^{\circ}}$ to $60^{{}^{\circ}}$. This trend reflects the expected behavior of the piezoelectric sensors, where higher angular displacement induces greater mechanical deformation, resulting in stronger electrical outputs.

For the female participant, the highest voltage output was recorded at $50^{{}^{\circ}}$ and $60^{{}^{\circ}}$, with values noticeably higher than those observed under the no-load condition (Figure 3). The most significant increase was observed between $20^{{}^{\circ}}$ and $30^{{}^{\circ}}$, which may indicate greater control and muscle engagement under load at moderate flexion ranges. In contrast, lower angles such as $10^{{}^{\circ}}$ exhibited less prominent differences between the no-load and 0.5 kg conditions, likely due to minimal strain generation at smaller joint excursions.

The male participant also exhibited elevated voltage outputs at all angles under load (Figure 7), with a sharper increase evident beyond $30^{{}^{\circ}}$. Compared to the no-load profile (Figure 4), the RMS values were significantly augmented, especially at $40^{{}^{\circ}}$ and $50^{{}^{\circ}}$, suggesting enhanced mechanical coupling between the sensor and skin-tissue interface during loaded flexion.

These results demonstrate that external loading has a measurable effect on the voltage output of the piezoelectric sensors, confirming their ability to reflect biomechanical stress and joint motion. The data also emphasize the system’s load sensitivity, making it a promising tool for dynamic monitoring in functional rehabilitation and strength training environments.

**Figure 8:**
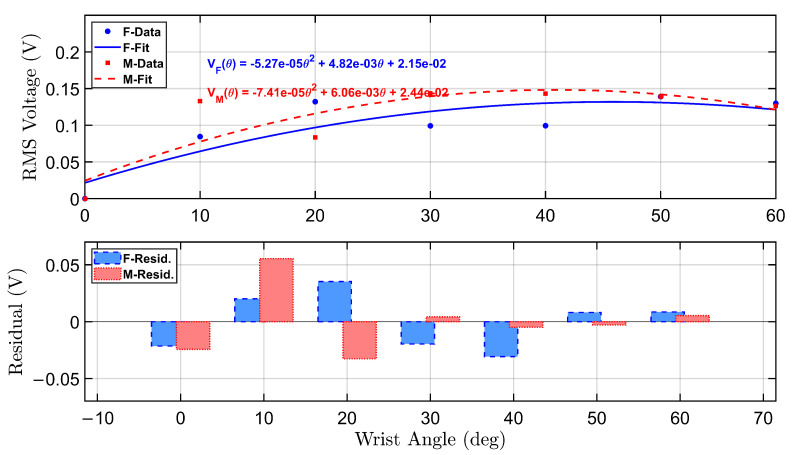
Calibration curves and residual errors for the smart wrist brace under a 0.5 kg load. Polynomial fits and error deviations are shown for both female and male participants across wrist angles from $0^{{}^{\circ}}$ to $60^{{}^{\circ}}$.

Figure 8 presents the calibration curves and corresponding residual errors for the smart wrist brace under a 0.5 kg load condition. The top plot shows second-order polynomial fits for both female and male participants, capturing the nonlinear relationship between wrist flexion angle and RMS voltage output. Compared to the no-load condition, the voltage response under load is amplified, particularly at intermediate angles, suggesting enhanced piezoelectric engagement due to the added resistance from the external weight.

The bottom plot displays the residual errors from the fitted curves. While the polynomial fits closely match the experimental data, minor deviations are observed primarily at lower and mid-range angles, indicating some variability in movement consistency or loading dynamics. The female residuals exhibit slightly larger fluctuations, potentially due to individual differences in muscle control or brace-skin interaction under load. Nonetheless, both curves maintain low residual magnitudes, validating the suitability of quadratic models for real-time calibration.

### 3.4. 1.0 kg Load Condition

**Figure 9:**
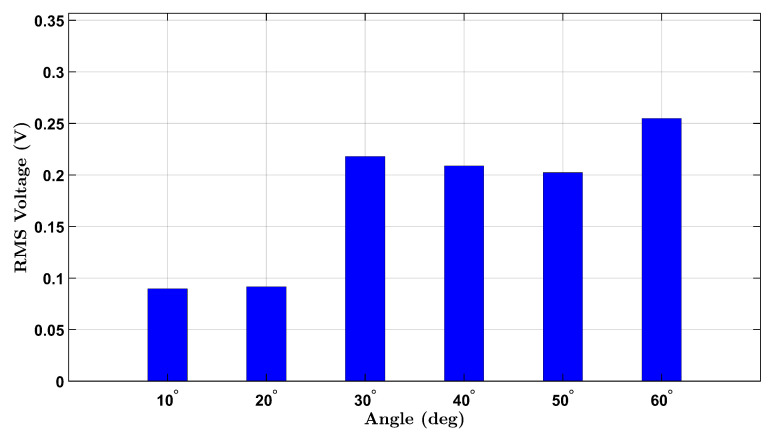
RMS voltage response of the female participant during wrist flexion under a 1.0 kg load across six target angles.

**Figure 10:**
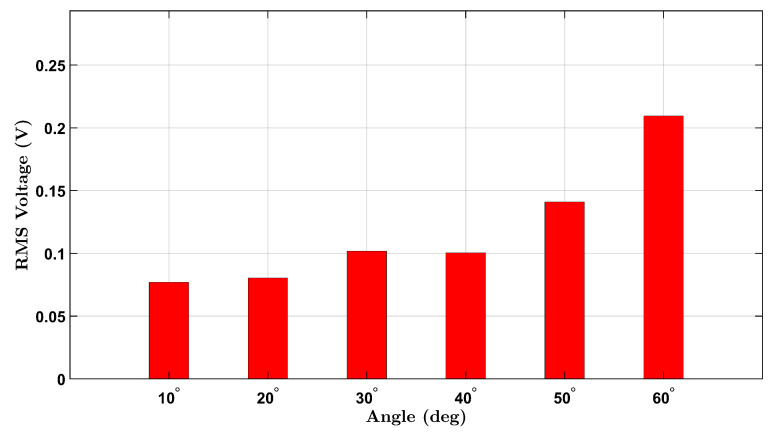
RMS voltage response of the male participant during wrist flexion under a 1.0 kg load across six target angles.

Figure 9 and Figure 10 display the RMS voltage responses measured from the female and male participants, respectively, during wrist flexion under a 1.0 kg external load. Each bar represents the average voltage magnitude generated by the piezoelectric sensor during repetitive wrist bending toward fixed target angles ranging from $10^{{}^{\circ}}$ to $60^{{}^{\circ}}$.

For the female participant (Figure 9), the RMS voltage exhibits a consistent and pronounced increase with the flexion angle, peaking at $60^{{}^{\circ}}$ with a value exceeding 0.25 V. This monotonic rise demonstrates that larger angular movements under heavier loading induce greater strain on the sensor, thereby producing stronger piezoelectric outputs. Compared to the 0.5 kg and no-load conditions, the 1.0 kg dataset shows a steeper slope and higher absolute values across all angles, indicating the system’s strong load sensitivity and the piezoelectric sensor’s effective response to mechanical deformation.

The male participant’s results (Figure 10) follow a similar trend, with RMS voltages increasing across the flexion range. The signal amplitude reaches approximately 0.21 V at $60^{{}^{\circ}}$, displaying a gradual but steady rise from lower angles. Notably, the voltage gains from 0.5 kg to 1.0 kg loads are more apparent in the male participant’s profile than in the transition from no load to 0.5 kg, suggesting a nonlinear relationship between applied load and mechanical strain. Additionally, while the male participant’s voltage values remain slightly lower than the female’s across most angles, the gap narrows under the 1.0 kg load, possibly due to anatomical or muscular differences that modulate sensor contact and wrist stiffness.

These results further support the hypothesis that the smart wrist brace system is capable of capturing biomechanical strain variations corresponding to both angular displacement and external loading. The proportional increases in RMS voltage values across all loading conditions confirm the system’s reliability for monitoring joint mechanics under resistance training or rehabilitative movements.

**Figure 11:**
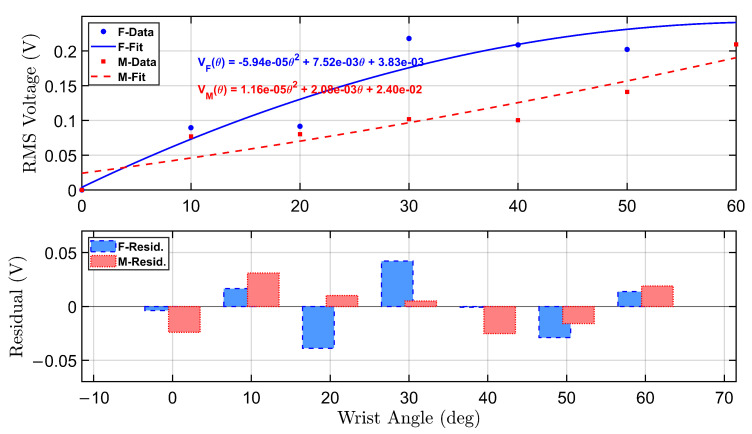
Polynomial calibration curves and residuals under 1.0 kg load for female (blue) and male (red) participants.

Figure 11 presents the polynomial calibration curves and corresponding residual errors for the female and male participants under a 1.0 kg load condition. The upper subplot illustrates second-order polynomial fits to the RMS voltage response at each wrist flexion angle. Both fits demonstrate a nonlinear relationship between angle and voltage, with the female curve exhibiting a more pronounced curvature, reflecting higher sensitivity to angular displacement at increased loads.

The fitted equations are shown in the top-right corner of the plot and are color-coded by participant. Notably, the female participant produced a higher voltage response across nearly all angles compared to the male, confirming consistent load-dependent sensor behavior.

The bottom subplot shows residual errors between the actual RMS voltage and the fitted curves. Most residuals remain within ±0.07 V, indicating good agreement between the measured and predicted values. However, slight systematic deviations appear at intermediate angles (20°–40°), particularly for the female participant, suggesting the presence of minor nonlinearities or subject-specific variations.

### 3.5. Comparative Analysis

**Figure 12:**
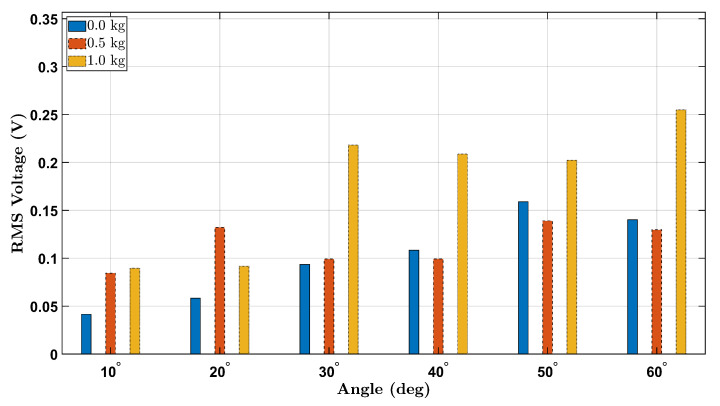
RMS voltage comparison across all load conditions (0.0 kg, 0.5 kg, 1.0 kg) for the female participant.

**Figure 13:**
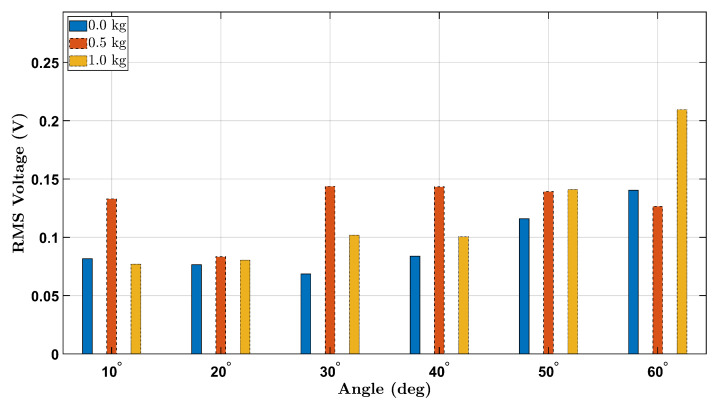
RMS voltage comparison across all load conditions (0.0 kg, 0.5 kg, 1.0 kg) for the male participant.

To further clarify the effect of external load on piezoelectric sensor output during wrist flexion, Figure 12 and Figure 13 present consolidated bar plots comparing the RMS voltage values for all three tested loads (0 kg, 0.5 kg, and 1.0 kg) across the six wrist flexion angles for the female and male participants, respectively.

The overall trend across both participants indicates that increasing the external load generally results in elevated RMS voltage output, especially at larger flexion angles (e.g., $50^{{}^{\circ}}$ and $60^{{}^{\circ}}$). This behavior is consistent with the mechanical principle that greater applied load amplifies strain on the wrist joint, which in turn increases the deformation of the integrated piezoelectric sensor and yields higher electrical output.

Notably, the female participant shows a more distinct voltage increase with load escalation, particularly between the 0.5 kg and 1.0 kg conditions at $30^{{}^{\circ}}$ to $60^{{}^{\circ}}$. This may suggest a stronger sensitivity to loading effects in her wrist biomechanics or a difference in muscle control strategy. In contrast, the male participant demonstrates a comparatively more uniform trend, with voltage values rising more moderately between 0.0 kg and 0.5 kg, and a clearer increase appearing only under the 1.0 kg condition at higher angles.

Interestingly, for angles below $30^{{}^{\circ}}$, the increase in RMS voltage with added load is less pronounced or inconsistent across participants. This may be attributed to the limited range of wrist flexion at lower angles, which produces relatively small strain gradients, leading to marginal differences in sensor activation regardless of load.

Together, these comparative figures offer a compelling visualization of how both mechanical angle and external loading interact to influence the dynamic behavior of the piezoelectric sensor system. These findings reinforce the importance of considering loading conditions when designing smart wearable systems for biomechanical assessment and injury prevention.

### 3.6. Strain Sensitivity Analysis

**Figure 14:**
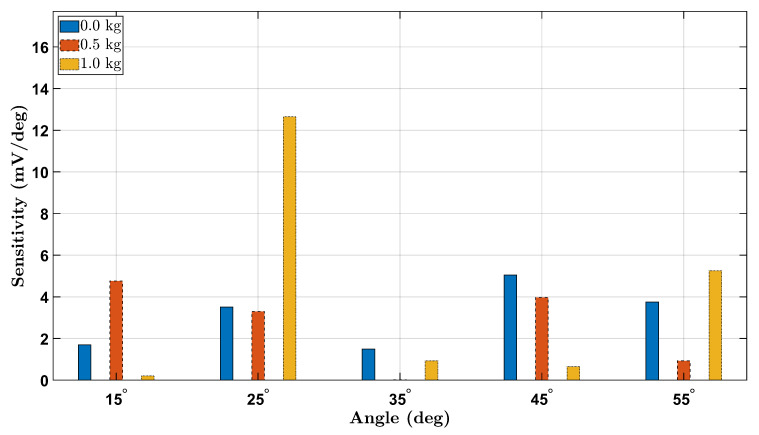
Strain sensitivity (mV/deg) for the female participant across all wrist angles and loads.

**Figure 15:**
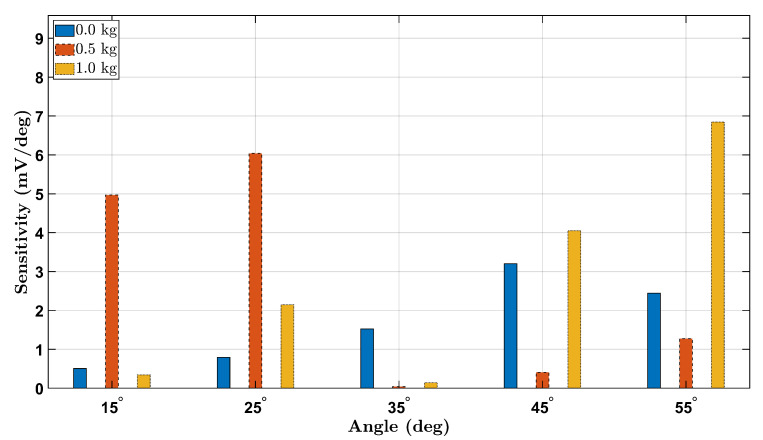
Strain sensitivity (mV/deg) for the male participant across all wrist angles and loads.

To evaluate the strain sensitivity of the piezoelectric sensing system across different wrist angles and loading conditions, we computed the voltage sensitivity as the change in RMS voltage per degree ($\frac{dV}{d\theta }$) between consecutive angle increments. Figure 14 and Figure 15 present the absolute sensitivity responses for the female and male participants, respectively, under 0 kg, 0.5 kg, and 1.0 kg loads.

The sensitivity trends reveal a general increase with load magnitude, highlighting the sensor’s enhanced response to higher mechanical strain. For both participants, sensitivity was highest at larger angles under the 1.0 kg load, confirming improved voltage resolution at elevated deformations. Notably, the female participant exhibited higher overall sensitivity values compared to the male, potentially due to anatomical and biomechanical differences. These findings demonstrate that the Smart Wrist Brace effectively amplifies signal detection with increasing wrist flexion and external loading, reinforcing its potential for dynamic monitoring of joint mechanics.

To comprehensively visualize the combined effects of wrist flexion angle and external load on the voltage output of the smart wrist brace, 3D surface plots were generated for both female and male participants. These plots illustrate the RMS voltage response as a function of wrist angle (θ) and load (0, 0.5, and 1.0 kg), capturing the continuous and interactive relationship between these two parameters.

**Figure 16:**
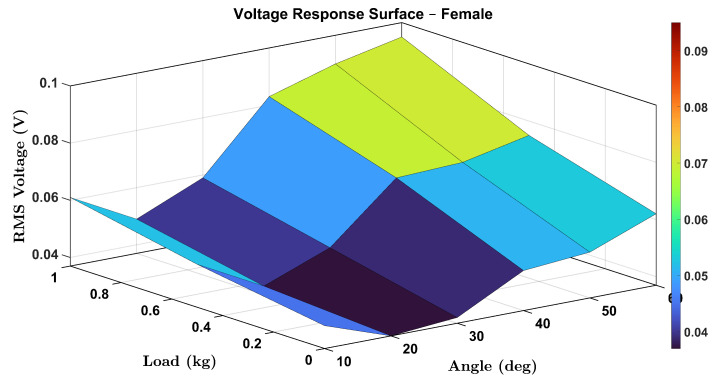
Three-dimensional (3D) surface plot showing the RMS voltage response of the female participant as a function of wrist angle and applied load.

Figure 16 displays the surface plot for the female participant. A clear upward trend in voltage response is observed with increasing angle and load, confirming that both parameters contribute synergistically to the strain-induced piezoelectric signal. Notably, the increase is more pronounced at higher angles, indicating nonlinear mechanical behavior likely associated with increased tendon and skin tension.

**Figure 17:**
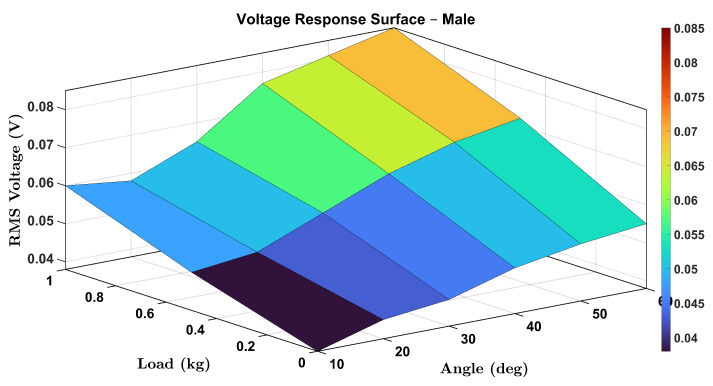
Three-dimensional (3D) surface plot showing the RMS voltage response of the male participant as a function of wrist angle and applied load.

Similarly, Figure 17 presents the male participant’s response surface. While the general trend remains consistent, voltage rising with both load and angle, the rate of increase appears slightly lower than in the female case, which could be attributed to physiological differences in muscle stiffness, wrist geometry, or skin elasticity.

These surface plots provide an intuitive visualization of system performance and further reinforce the robustness and sensitivity of the proposed sensing mechanism. The 3D mapping also enables predictive modeling of voltage outputs under new or interpolated loading/angle combinations, supporting real-time monitoring applications.

## 4. Lumped Parameter Modeling

**Figure 18:**
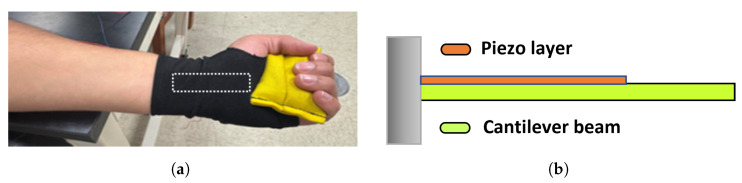
Schematic representation of the lumped-parameter modeling approach. (**a**) The wrist–hand structure during flexion. (**b**) Mechanical approximation using a cantilever beam with a bonded piezoelectric layer subjected to bending-induced strain.

To mechanistically interpret the voltage outputs measured during wrist flexion, we introduce a simplified lumped-parameter mechanical model of the wrist–hand assembly. As illustrated in Figure 18, the system is approximated as a cantilever beam subjected to tip deflection, where the beam represents the hand and a surface-bonded piezoelectric film acts as the sensing layer. This modeling approach provides analytical tractability while capturing the essential mechanical behavior responsible for strain-induced voltage generation across the sensor.

### 4.1. Mechanical Approximation Using a Cantilever Beam

The wrist brace structure under bending resembles a cantilever beam of length *L*, fixed at one end (the wrist joint) and deflected at the free end (the hand). When the user bends the wrist by an angle θ, the vertical tip displacement δ is given by:

(1) δ=Lsin(θ)≈Lθforsmallangles(θinradians).

Assuming linear elastic behavior, the effective restoring force at the tip is:

(2) F=kδ=kLθ,

where *k* is the equivalent stiffness of the wrist-brace system.

This force induces a bending moment at the base of the beam (wrist joint) as:

(3) $$M=FL=kL^{2}\theta .$$

### 4.2. Piezoelectric Voltage Response Modeling

The surface-bonded piezoelectric film experiences mechanical strain due to curvature induced by wrist flexion. The curvature κ of the beam at the base is approximated as:

(4) $$\kappa =\frac{\partial^{2}w}{\partial x^{2}}\approx \frac{\theta }{L}.$$

Under the direct piezoelectric effect, this curvature translates into a voltage output given by:

(5) $$V\left(\theta \right)=d_{31}Yh\kappa L_{\mathrm{pz}}=\underset{\beta }{\underset{︸}{d_{31}Yh\cdot L_{\mathrm{pz}}\cdot \frac{1}{L}}}\cdot \theta ,$$

where $d_{31}$ represents the piezoelectric strain constant, quantifying the electric field generated per unit mechanical strain. The term *Y* denotes the Young’s modulus of the piezoelectric layer, capturing its stiffness. The parameter *h* refers to the thickness of the piezoelectric film, while $L_{\mathrm{pz}}$ is the effective length over which the piezoelectric sensor is bonded to the beam. Together, these parameters define the voltage sensitivity coefficient β, which governs the linear relationship between wrist angle and output voltage and is expressed in units of volts per degree (V/deg). This relation simplifies to the below equation for capturing the directly proportional behavior between bending angle and voltage response.

(6) V(θ)=βθ,

In this study, the piezoelectric layer thickness *h* was fixed at 52 μm, as a commercially available PVDF film was used consistently across all experiments. No thickness variation was studied, and the parameter *h* is treated as a constant in the theoretical model. Furthermore, due to the film’s small thickness relative to the overall wrist brace structure, its influence on the global mechanical stiffness is considered negligible.

### 4.3. Model Calibration and Validation

The linear model above was calibrated using experimental RMS voltage values recorded across known wrist angles for both participants under the no-load condition. Using least-squares regression, the following empirical linear fits were extracted:

(7) $$\begin{array}{cc} V_{F}\left(\theta \right) & =a_{F}\;\theta +b_{F}, \end{array}$$

(8) $$\begin{array}{cc} V_{M}\left(\theta \right) & =a_{M}\;\theta +b_{M}, \end{array}$$

where $V_{F}$ and $V_{M}$ represent the female and male voltage outputs in volts, and θ is the wrist angle in degrees. Also, $a_{F}$ and $a_{M}$ represent the sensitivity coefficients (in V/deg) for the female and male participants, respectively, indicating the rate of change in voltage output with respect to wrist angle. The constants $b_{F}$ and $b_{M}$ denote the voltage offsets at zero angle, capturing any baseline voltage present due to sensor pre-strain, system bias, or fabrication inconsistencies. These coefficients were obtained through linear regression of experimental data collected under the no-load condition.

These results indicate that the piezoelectric voltage response can be modeled with high fidelity using a linear formulation, with the slope β reflecting the effective voltage sensitivity of the brace–sensor system.

### 4.4. Comparison with Experimental Data

**Figure 19:**
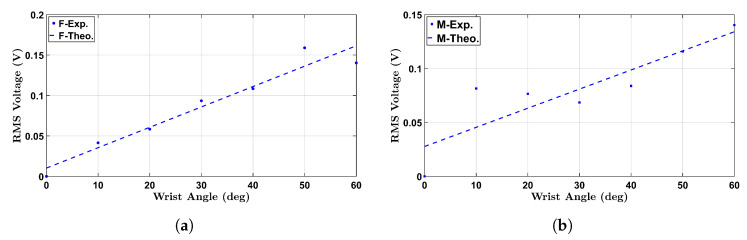
Model validation under no-load condition: (**a**) Female participant, (**b**) Male participant. Solid markers represent experimental RMS voltage data, and dashed lines indicate the fitted theoretical predictions based on a linear model.

To validate the proposed lumped-parameter model, we compared the experimentally measured RMS voltage outputs with the theoretical predictions obtained from the fitted linear equations. Figure 19 shows the comparison for both the female and male participants under the no-load condition. The results confirm strong agreement between the experimental and model-based voltage responses, particularly in the $10^{{}^{\circ}}$ to $60^{{}^{\circ}}$ range, with deviations mainly observed at low-angle motion due to signal noise and measurement sensitivity.

For the no-load condition, the fitted calibration equations used for the theoretical predictions were:

(9) $$\begin{array}{cc} V_{F}\left(\theta \right) & =\beta_{F,0}\theta +b_{F,0}, \end{array}$$

(10) $$\begin{array}{cc} V_{M}\left(\theta \right) & =\beta_{M,0}\theta +b_{M,0}, \end{array}$$

where $\beta_{F,0}=0.00259$ V/deg and $b_{F,0}=-0.0060$ V for the female participant, while $\beta_{M,0}=0.00240$ V/deg and $b_{M,0}=-0.0066$ V for the male participant. These coefficients reflect the sensor’s angular sensitivity and baseline offset, respectively.

Table 1 summarizes the extracted coefficients from linear fits of the form V(θ)=aθ+b for both female and male participants under all three loading conditions (0.0 kg, 0.5 kg, and 1.0 kg). These coefficients offer insight into the load-dependent sensitivity of the piezoelectric voltage generation.

The results clearly demonstrate that the slope *a* (representing the voltage sensitivity per degree) increases with load for both participants, indicating enhanced strain transmission to the piezoelectric layer under higher mechanical loading. The intercept *b* progressively shifts toward zero, reflecting improved contact and reduced slack in the sensor-brace system.

While the calibration curves derived experimentally employed second-order polynomial fits to capture slight nonlinearities in the piezoelectric voltage response, the lumped-parameter mechanical model fundamentally predicts a linear relationship between wrist flexion angle and voltage generation. This apparent discrepancy is minor and arises primarily due to experimental factors such as viscoelasticity of the skin-brace interface, minor fabrication inconsistencies, or hysteresis effects at larger angular deformations. The strong agreement observed between the model-predicted linear voltage trends and experimental data across all loading conditions supports the validity of the simplified theoretical assumptions. The minor nonlinearities observed experimentally are consistent with expected real-world biomechanical behavior and do not contradict the fundamental model, but rather highlight opportunities for further refinement in future wearable sensing designs.

## 5. Final Remarks and Future Outlook

The findings presented in this study reinforce the value of self-powered wearable systems for biomechanical monitoring during dynamic human motion. The smart wrist brace, integrating piezoelectric sensing and simplified mechanical modeling, not only enables real-time tracking of wrist flexion under varied loading but also demonstrates sensitivity to subject-specific biomechanical differences. The consistency of voltage responses, the robustness of the linear and polynomial calibration models, and the clarity of residual and surface analyses validate the system’s ability to bridge experimental observations with predictive modeling. Collectively, these results support the practical deployment of this technology in real-world scenarios, including personalized rehabilitation, athlete training, and ergonomic assessment.

The voltage–angle relationship established in this study lays the groundwork for implementing intelligent data-driven models in future iterations of the smart wrist brace. Specifically, machine learning techniques such as support vector machines (SVM), decision trees, or neural networks could be trained on labeled voltage–angle datasets to perform real-time classification of motion patterns or regression-based joint angle estimation. Such models could enhance the system’s ability to detect improper lifting techniques or fatigue-induced anomalies, making it more responsive for rehabilitation and athletic training applications.

## 6. Conclusions

This work introduces and validates a self-powered smart wrist brace equipped with integrated piezoelectric sensing for continuous biomechanical monitoring during weightlifting tasks. The system demonstrated effective strain-to-voltage transduction across six wrist angles and three loading conditions for both male and female users. Experimental data revealed clear voltage–angle–load dependencies, while calibration models and residual analyses confirmed strong predictive capabilities through second-order and linear polynomial fits. A lumped-parameter mechanical model, based on a cantilever beam analogy, was used to interpret the voltage generation mechanism and was further supported by theoretical simulations and experimental comparisons. The extracted sensitivity coefficients increased with external load, confirming the device’s load-responsiveness.

Strain sensitivity analyses and 3D surface mapping further highlighted the system’s ability to differentiate between users and loading conditions, offering pathways for personalization and real-time motion classification. By bridging wearable sensing, biomechanics, and theoretical modeling, this work lays the foundation for practical applications in athlete training, rehabilitation, and ergonomic assessment. Future work will focus on expanding the sample size, subject trials, integrating wireless modules, and deploying machine learning algorithms for real-time motion recognition and predictive feedback in clinical and sports environments.

## Figures and Tables

**Table 1:** Linear fit coefficients *a* and *b* for V(θ)=aθ+b.

Load (kg)	Female Participant		Male Participant	
	$a_{F}$ (V/deg)	$b_{F}$ (V)	$a_{M}$ (V/deg)	$b_{M}$ (V)
0.0	0.00259	−0.0060	0.00240	−0.0066
0.5	0.00281	−0.0042	0.00261	−0.0043
1.0	0.00302	−0.0025	0.00288	−0.0021

## Data Availability

Data available on request from the authors.
